# Supplementary material for: Cross-sectional analysis of national testosterone prescribing through prescription drug monitoring programs, 2018–2022
Source: PLoS One. 2024 Aug 28;19(8):e0309160. doi: 10.1371/journal.pone.0309160 (PMC11355536; doi:10.1371/journal.pone.0309160)
Supplement: Attachment — Submitted filename: IRB approval letter.pdf [file pone.0309160.s002.pdf]

The University of Texas at Austin

Office of Research Support & Compliance
Institutional Review Board
P.O. Box 7426, Campus Code A3200
Austin, Texas 78713
T: 512-232-1543 F: 512-471-8873
Email: irb@austin.utexas.edu
www.research.utexas.edu/ors

NOT HUMAN RESEARCH

October 21, 2022

Scott Selinger
1601 TRINITY ST BLDG A
AUSTIN, TX 78712

s9344@eid.utexas.edu

Dear Scott Selinger:

On 10/21/2022, the IRB reviewed the following submission:

Type of Review:	Initial Study
Title of Study:	Testosterone Rx patterns - NHSR determination
Investigator:	Scott Selinger
IRB ID:	STUDY00003594
Funding:	None
Grant Title:	None
Grant ID:	None
IND, IDE, or HDE:	None
Documents Reviewed:	• NHSR request , Category: IRB Protocol;

The IRB determined that the proposed activity is not research involving human subjects as defined by DHHS and FDA regulations. The activity was determined to be Research using publicly available, de-identified, secondary use data set.

IRB review and approval by this organization is not required. This determination applies only to the activities described in the IRB submission and does not apply should any changes be made. If you have any additional questions regarding whether future activities would be considered human subject research, please contact the Office of Research Support and Compliance at irb@austin.utexas.edu.

Sincerely,

Institutional Review Board

The University of Texas at Austin

Office of Research Support & Compliance
Institutional Review Board
P.O. Box 7426, Campus Code A3200
Austin, Texas 78713
T: 512-232-1543 F: 512-471-8873
Email: irb@austin.utexas.edu
www.research.utexas.edu/ors

University of Texas at Austin

cc:
Scott Selinger (PI), Katherine Sebastian (Primary Contact)

Template Revision: June 8, 2020
