# Supplementary material for: Cross-sectional analysis of national testosterone prescribing through prescription drug monitoring programs, 2018–2022
Source: PLoS One. 2024 Aug 28;19(8):e0309160. doi: 10.1371/journal.pone.0309160 (PMC11355536; doi:10.1371/journal.pone.0309160)
Supplement: Attachment — Submitted filename: Response to Reviewers.docx [file pone.0309160.s004.docx]

**Response to Reviewers – Manuscript ID: PONE-D-24-13515**

“Cross-Sectional Analysis of National Testosterone Prescribing through Prescription Drug Monitoring Programs, 2018-2022”

**Reviewer #1**

| **#** | **Comments** | **Authors’ Response** |
| --- | --- | --- |
| 1 | I'm still not very convinced that in Figure 4 it is reported incidence. The values seems very similar to that reported in table 3 (total column). This means that almost all TT users were "new users"? In any case, I would like to suggest to better explain in the methods section (line 87, page 11) how incident users were defined (subjects with a TT claim in a year and with no TT claims in the previous one?) | *Thank you for your keen eye. It was an incorrect use of the phrase incidence rate ratio. We have changed this to simple population prevalence and changed the figure and text as such as there was no real way to identify incident users in the aggregated datasets used.* |
